# Supplementary material for: Impact of Ar Flow Rates on Micro-Structural Properties of WS2 Thin Film by RF Magnetron Sputtering
Source: Nanomaterials (Basel). 2021 Jun 22;11(7):1635. doi: 10.3390/nano11071635 (PMC8306877; doi:10.3390/nano11071635)
Supplement: Supplementary file 1 [file nanomaterials-11-01635-s001.zip › nanomaterials-1260805-supplementary.pdf]

nanomaterials

Supporting Information

Impact of Ar Flow Rates on Micro-Structural Properties of WS_2 Thin Film by RF Magnetron Sputtering

Md. Akhtaruzzaman [1], M. Shahiduzzaman [2,*], N. Amin [3,*], Ghulam Muhammad [4], Mohammad Aminul Islam [5], K. Sobayel [1,*] and K. Sopian [1]

[1] Solar Energy Research Institute, The National University of Malaysia, Bangi 43600, Selangor, Malaysia; akhtar@ukm.edu.my (M.A.); ksopian@ukm.edu.my (K.S.)
[2] Graduate School of Natural Science and Technology, Kanazawa University, Kakuma, Kanazawa 920-1292, Japan
[3] Institute of Sustainable Energy, Universiti Tenaga Nasional (@The National Energy University), Jalan Ikram-Uniten, Kajang 43000, Selangor, Malaysia
[4] Department of Computer Engineering, College of Computer and Information Sciences, King Saud University,11451 Riyadh, Saudi Arabia; ghulam@ksu.edu.sa
[5] Department of Electrical Engineering, University of Malaya, Jalan Universiti, Kuala Lumpur 50603, Malaysia; aminul.islam@um.edu.my
* Correspondence: shahiduzzaman@se.kanazawa-u.ac.jp (M.S.); nowshad@uniten.edu.my (N.A.); sobayel@ukm.edu.my (K.S.)

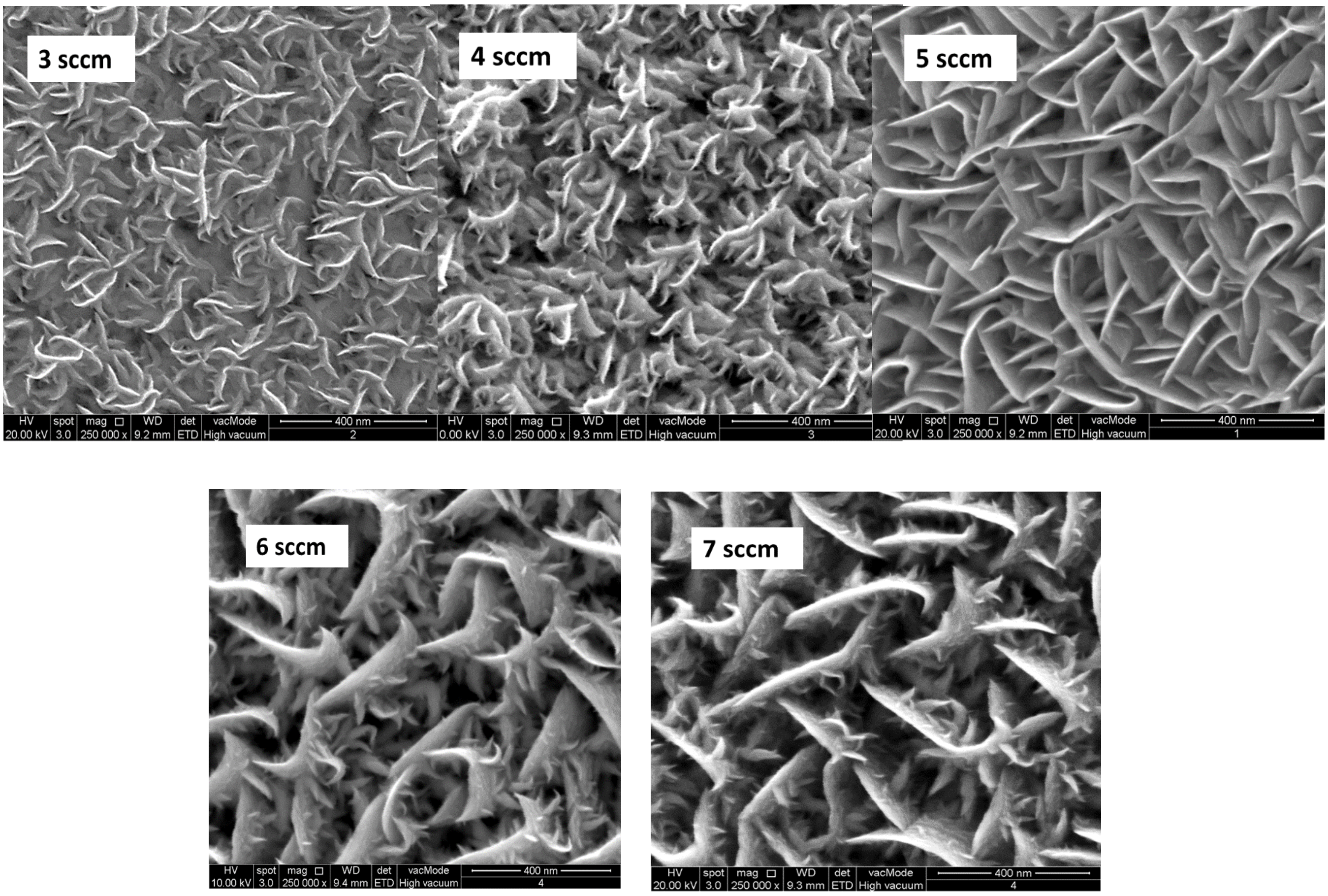

Figure S1. FESEM images of WS_2.

Table S1. Thickness of WS_2 thin film.

Gas flow rate(sccm)	Thickness(nm)
**3**	159.4
**4**	167.8
**5**	170.7
**6**	123.9
**7**	101.2

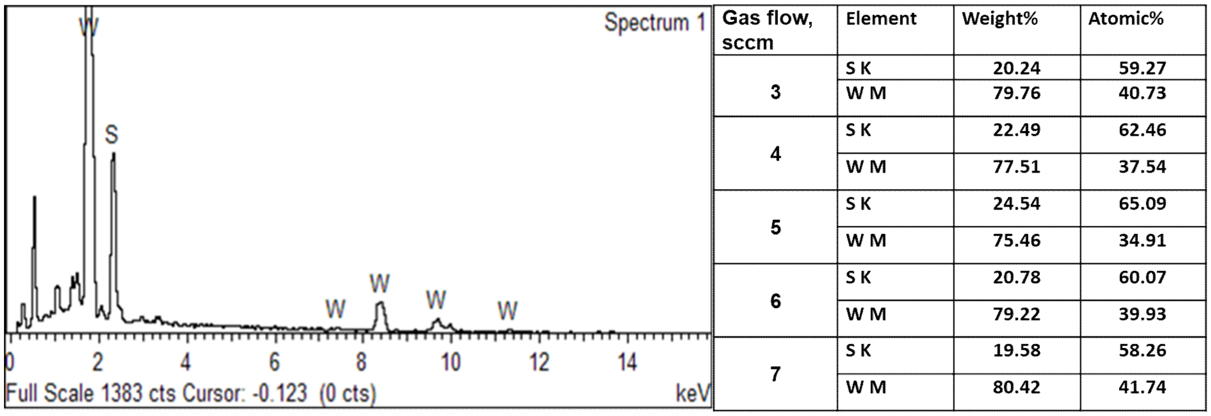

Gas flow, sccm	Element	Weight%	Atomic%
3	S K	20.24	59.27
	W M	79.76	40.73
4	S K	22.49	62.46
	W M	77.51	37.54
5	S K	24.54	65.09
	W M	75.46	34.91
6	S K	20.78	60.07
	W M	79.22	39.93
7	S K	19.58	58.26
	W M	80.42	41.74

Figure S2. EDX data of WS2 thin film under various Ar flow rate.

Table S2: Contact Material Properties

**Property**	**Front contact**	**Back contact**	
Surface recombination velocity of electron	10^7 cm/s	10^5 cm/s	10^5 cm/s
Surface recombination velocity of hole	10^5 cm/s	10^7 cm/s	10^7 cm/s
Metal work function	Flat band	Flat band	Mo (5 eV)

Table S3. Material Properties Used for simulation [1–5].

Parameters	CdTe	WS_2	ZnO
Thickness (nm)	1500	100	100
E_g (eV)	1.5	2.1	3.3
χ (eV)	4.6	3.95	4.40
ε_r	9.4	13.6	9
N_c (cm^{-3})	2.2×10^{18}	2.0×10^{18}	2.0×10^{18}
N_v (cm^{-3})	1.5×10^{19}	2.0×10^{18}	1.8×10^{19}
μ_e (cm^2/Vs)	100	100	100
μ_h (cm^2/Vs)	12.5	25	25
N_D (cm^{-3})	-	Experiment	1.0×10^{18}
N_A (cm^{-3})	2.0×10^{17}	–	-

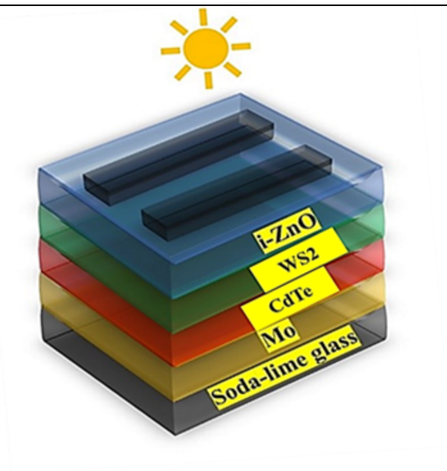

Figure S3. Schematic of CdTe/WS_2 solar cell.

Reference

1. Bin Rafiq, M.K.S., Amin, N., Alharbi, H.F et al. WS_2: A New Window Layer Material for Solar Cell Application. *Sci. Rep.* **2020**, *10*, 771, doi:10.1038/s41598-020-57596-5.
2. M. A. Islam, K. Sobayel et al. Tailoring of the Structural and Optoelectronic Properties of Zinc-Tin-Oxide Thin Films via Oxygenation Process for Solar Cell Application. *IEEE Access* **2020**, *8*, 193560-193568, doi:10.1109/ACCESS.2020.3031894.
3. S. Mahjabin, Md Mahfuzul Haque, K. Sobayel et al. Perceiving of Defect Tolerance in Perovskite Absorber Layer for Efficient Perovskite Solar Cell. *IEEE Access* **2020**, *8*, 106346-106353, doi: 10.1109/ACCESS.2020.3000217.
4. K. Sobayel, Md. Akhtaruzzaman, et.al. A comprehensive defect study of tungsten disulfide (WS2) as electron transport layer in perovskite solar cells by numerical simulation. *Results in Physics* **2019**, *12*, 1097-2003.
5. F.M.T. Enam, K.S. Rahman, M.I. Kamaruzzaman, K. Sobayel, B. Bais, M. Akhtaruzzaman, A.R.M. Alamoud & N.Amin. 2016. Design Prospects of Cadmium Telluride/Silicon (CdTe/Si) Tandem Solar Cells from Numerical Simulation. *Optik* **2016**, *17* 376-379.
